# Supplementary material for: Adversarial attacks and adversarial robustness in computational pathology
Source: Nat Commun. 2022 Sep 29;13:5711. doi: 10.1038/s41467-022-33266-0 (PMC9522657; doi:10.1038/s41467-022-33266-0)
Supplement: Supplementary file 2 — Reporting Summary [file 41467_2022_33266_MOESM2_ESM.pdf]

nature portfolio

Corresponding author(s): JN Kather

Last updated by author(s): Sep 6, 2022

Reporting Summary

Nature Portfolio wishes to improve the reproducibility of the work that we publish. This form provides structure for consistency and transparency in reporting. For further information on Nature Portfolio policies, see our Editorial Policies and the Editorial Policy Checklist.

Statistics

For all statistical analyses, confirm that the following items are present in the figure legend, table legend, main text, or Methods section.

n/a	Confirmed	
☐	☒	The exact sample size (*n*) for each experimental group/condition, given as a discrete number and unit of measurement
☐	☒	A statement on whether measurements were taken from distinct samples or whether the same sample was measured repeatedly
☐	☒	The statistical test(s) used AND whether they are one- or two-sided<br>*Only common tests should be described solely by name; describe more complex techniques in the Methods section.*
☒	☐	A description of all covariates tested
☐	☒	A description of any assumptions or corrections, such as tests of normality and adjustment for multiple comparisons
☐	☒	A full description of the statistical parameters including central tendency (e.g. means) or other basic estimates (e.g. regression coefficient) AND variation (e.g. standard deviation) or associated estimates of uncertainty (e.g. confidence intervals)
☐	☒	For null hypothesis testing, the test statistic (e.g. *F*, *t*, *r*) with confidence intervals, effect sizes, degrees of freedom and *P* value noted<br>*Give P values as exact values whenever suitable.*
☒	☐	For Bayesian analysis, information on the choice of priors and Markov chain Monte Carlo settings
☐	☒	For hierarchical and complex designs, identification of the appropriate level for tests and full reporting of outcomes
☒	☐	Estimates of effect sizes (e.g. Cohen's *d*, Pearson's *r*), indicating how they were calculated

Our web collection on statistics for biologists contains articles on many of the points above.

Software and code

Policy information about availability of computer code

Data collection	No specific software was used to collect the data. Data preprocessing is described in "Data Analysis".
Data analysis	All source codes are publicly available: for image preprocessing, codes are available at https://github.com/KatherLab/preProcessing; for the baseline image analysis, codes are available at https://github.com/KatherLab/HIA and for adversarial attacks, codes are available at https://github.com/ KatherLab/Pathology_Adversarial. In this work, we used Python 3.10.6, Pytorch (1.11.0+cu113), Scikit-learn (1.1.2), Numpy (1.23.1), Pandas (1.4.3), OpenCV (4.6.0), pytorch_pretrained_vit (0.0.7), pickle (0.7.5), efficientnet_pytorch (0.7.1) and torchvision (0.12.0 +cu113).

For manuscripts utilizing custom algorithms or software that are central to the research but not yet described in published literature, software must be made available to editors and reviewers. We strongly encourage code deposition in a community repository (e.g. GitHub). See the Nature Portfolio guidelines for submitting code & software for further information.

Data

Policy information about availability of data

All manuscripts must include a data availability statement. This statement should provide the following information, where applicable:

- Accession codes, unique identifiers, or web links for publicly available datasets
- A description of any restrictions on data availability
- For clinical datasets or third party data, please ensure that the statement adheres to our policy

The data that support the findings of this study are mostly publicly available, in part proprietary datasets provided under collaboration agreements. All data

(including histological images) from the TCGA database are available at https://portal.gdc.cancer.gov/. The cohort accession codes are TCGA-KIRC, TCGA-KIRP, TCGA-KICH and TCGA-STAD. Access to the proprietary data can be requested from the respective study groups who independently manage data access for their study cohorts: Rupert Langer for BERN-GASTRIC, Roman D. Buelow and Peter Boor for AACHEN-RCC. The respective principal investigators will decide within a reasonable timeframe if the data can be shared for research purposes under a dedicated collaboration agreement signed by the respective institution.

Human research participants

Policy information about studies involving human research participants and Sex and Gender in Research.

Reporting on sex and gender	Not applicable for this technical study.
Population characteristics	Not applicable for this technical study.
Recruitment	This technical study used retrospective tissue samples which were available through public repositories and provided as part of proprietary tissue collections at participating pathology departments. No inclusion or exclusion criteria for participan
Ethics oversight	This study was performed in accordance with the Declaration of Helsinki. We performed a retrospective analysis of anonymized patient samples. In addition to publicly available data from "The Cancer Genome Atlas" (TCGA, https://portal.gdc.cancer.gov), we used a renal cell carcinoma dataset by the University of Aachen, Germany (ethics board of Aachen University Hospital, No. EK315/19) and a gastric cancer dataset by the University of Bern (ethics board at the University of Bern, Switzerland, no. 200/14). This study adheres to the MI-CLAIM [50] checklist (Suppl. Table 1).

Note that full information on the approval of the study protocol must also be provided in the manuscript.

Field-specific reporting

Please select the one below that is the best fit for your research. If you are not sure, read the appropriate sections before making your selection.

☒ Life sciences ☐ Behavioural & social sciences ☐ Ecological, evolutionary & environmental sciences

For a reference copy of the document with all sections, see nature.com/documents/nr-reporting-summary-flat.pdf

Life sciences study design

All studies must disclose on these points even when the disclosure is negative.

Sample size	For renal cell carcinoma (RCC), two patient cohorts were used: TCGA-RCC (N=897 patients, Suppl. Figure 1A), and AACHEN-RCC (N=249, Suppl. Figure 1B). For gastric cancer, two patient cohorts were used: TCGA-GASTRIC (N=191 patients, Suppl. Figure 1C) and BERN-GASTRIC (N=249 patients, Suppl. Figure 1D). No formal method to calculate sample size for deep learning biomarker studies is available. The sample size was chosen according to sample availability and it was comparable to previous studies in the same field of research (e.g. Ghaffari Laleh et al., Medical Image Analysis, 2022)
Data exclusions	Reasons for data exclusion were missing image data, missing metadata and faulty image files resulting in pre-processing dropout. All dropouts are listed in Suppl. Figure 1.
Replication	We repeated all experiments five times with different random seeds. All attempts at replicating were successful and are reported in the manuscript.
Randomization	Random seeds for neural network training were generated with Python's random number generator. There were no randomized experimental groups in this study.
Blinding	As part of our study, we performed a reader study in which an expert observer evaluated image tiles. This observer was blinded during this evaluation. No other parts of our study required blinding.

Reporting for specific materials, systems and methods

We require information from authors about some types of materials, experimental systems and methods used in many studies. Here, indicate whether each material, system or method listed is relevant to your study. If you are not sure if a list item applies to your research, read the appropriate section before selecting a response.

Materials & experimental systems

n/a	Involved in the study
☒	☐ Antibodies
☒	☐ Eukaryotic cell lines
☒	☐ Palaeontology and archaeology
☒	☐ Animals and other organisms
☐	☒ Clinical data
☒	☐ Dual use research of concern

Methods

n/a	Involved in the study
☒	☐ ChIP-seq
☒	☐ Flow cytometry
☒	☐ MRI-based neuroimaging

Clinical data

Policy information about clinical studies

All manuscripts should comply with the ICMJEguidelines for publication of clinical research and a completedCONSORT checklist must be included with all submissions.

Clinical trial registration	No clinical trial was performed. In this study, we retrospectively used consecutive data from the routine clinical database.
Study protocol	No formal study protocol is available. The main analysis steps follow a procedure which has been previously established by Kather et al., Nature Medicine, 2019 (DOI 10.1038/s41591-019-0462-y).
Data collection	We collected digital whole slide images (WSI) of H&E-stained slides of archival tissue sections of human cancer from four patient cohorts.
Outcomes	The primary endpoint for this study was the area under the receiver operator characteristic curve (AUROC) for detection of categorical outputs. The AUROCs of five training runs of a given model were compared. A two-tailed unpaired t-test with p<= 0.05 was considered statistically significant. In the manuscript, AUROCs are given as mean +/- standard deviation.
